# Complete Genome Characterization of *Penicillimonavirus gammaplasmoparae*, a Bipartite Member of the Family *Mymonaviridae*

**DOI:** 10.3390/plants12183300

**Published:** 2023-09-18

**Authors:** Félix Morán, Antonio Olmos, Thierry Candresse, Ana Belén Ruiz-García

**Affiliations:** 1 Centro de Protección Vegetal y Biotecnología, Instituto Valenciano de Investigaciones Agrarias (IVIA), Ctra. Moncada-Náquera km 4.5, 46113 Valencia, Spain; moran_fel@gva.es (F.M.); olmos_antcas@gva.es (A.O.); 2 UMR 1332 Biologie du Fruit et Pathologie, INRAE, University Bordeaux, CEDEX, 33882 Villenave d’Ornon, France; thierry.candresse@inrae.fr

**Keywords:** grapevine, HTS, mymonavirus, *Penicillimonavirus gammaplasmoparae*, *Erysiphe necator*

## Abstract

In this study, we identified Plasmopara-viticola-lesion-associated mononegaambi virus 3 (recently classified as *Penicillimonavirus gammaplasmoparae*), a fungi-associated mymonavirus, in grapevine plants showing an unusual upward curling symptomatology on the leaves and premature decline. *Mymonaviridae* is a family comprising nine genera of negative-sense single-stranded RNA viruses infecting filamentous fungi, although few of them have been associated with oomycetes, plants, and insects. Although the first mymonavirus genome description was reported a decade ago, the genome organization of several genera in the family, including the genus *Penicillimonavirus,* has remained unclear to date. We have determined the complete genome of *P. gammaplasmoparae,* which represents the first complete genomic sequence for this genus. Moreover, we provide strong evidence that *P. gammaplasmoparae* genome is bipartite and comprises two RNA molecules of around 6150 and 4560 nt. Our results indicate that the grapevine powdery mildew pathogen, *Erysiphe necator,* was also present in the analyzed plants and suggest *P. gammaplasmoparae* could be infecting this fungus. However, whether the fungus and/or the mycovirus are associated with the symptomatology that initially prompted these efforts remains to be determined.

## 1. Introduction

Grapevine (*Vitis vinifera* L.) is one of the most important crops cultivated worldwide, with its efficient and sustainable production seen as a major goal for the agriculture and industry systems of many countries. Grapevine plants can be infected by many different pathogens that may cause important losses in grape quality and production, thus threatening viticulture. Among them, 35 viral species have been associated with major grapevine diseases, like infectious degeneration and decline, leafroll, rugose wood, fleck, red-blotch, leaf mottling and deformation, vein clearing, and Roditis leaf discoloration [1,2]. However, a much more complex grapevine virome has been described, with 97 viral species known from this crop to date [2,3,4,5,6,7,8,9].

High-throughput sequencing (HTS) technologies have heavily contributed to the exponential increase in plant virome knowledge achieved during the last two decades [10,11,12,13,14,15]. The main advantage of HTS-based approaches is their ability to identify unknown viruses even when present at low viral concentrations [11,15]. Different HTS approaches generate metagenomic data, which can improve our understanding of the complex microbiome associated with plants, including viruses that infect organisms associated with plants, such as fungi, bacteria, or insects [16]. In this scenario, new challenges arise for the understanding of the biological significance of the identification of a novel virus. To this end, viral genomic data can be complemented with additional evidence obtained from phylogenetic analyses, morphological characterization of virions, and host range studies.

*Mymonaviridae* is a recently described virus family, which comprises nine genera *Auricularimonavirus*, *Botrytimonavirus*, *Lentimonavirus*, *Penicillimonavirus*, *Sclerotimonavirus*, *Hubramonavirus*, *Phyllomonavirus*, *Plasmopamonavirus*, and *Rhizomonavirus* and 50 species [17]. Although most *Mymonaviridae* species have been reported infecting fungi, several species like Apple virus B, Gisinge virus, Kiln Barn virus, and Hubei rhabdo-like virus 4 have been found associated with plants or insects [18,19,20], suggesting that mymonaviruses hosts may not be limited to fungi.

The typical mymonavirus genome organization was described based on the first species reported, *Sclerotimonavirus sclerotinae* [21], and consists of a single molecule of negative-sense RNA of about 10 kilobases (kb) harboring six non-overlapping open reading frames (ORFs) encoding from 5′ to 3′ the following proteins: pI, nucleoprotein (NP), pIII, pIV, L protein corresponding to the virus RNA-dependent RNA polymerase (RdRp) and pVI. The ORFs are separated by non-coding intergenic regions (IGRs), which contain highly conserved gene junction sequences [17]. Other reported mymonaviruses genomes range between 6.2 and 11.6 kb and encode a variable number of ORFs (from four to seven), but some of the reported genomes are incomplete [17]. The precise genome size and number of ORFs for several genera (*Hubramonavirus, Penicillimonavirus, Plasmopamonavirus, Phyllomonavirus,* and *Rhizomonavirus*) are therefore unknown since the available genomic sequences are incomplete. No bipartite genomes have been described in the *Mymonaviridae* family to date.

The present study was initiated in an effort to understand the etiology of an unusual grapevine leafroll syndrome. A total RNAseq analysis showed the presence in these plants of *Penicillimonavirus gammaplasmoparae,* a recently identified fungi-associated mymonavirus species. Further experiments demonstrated that the genome of this virus is bipartite, and we report here the first complete genomic sequence of *Penicillimonavirus gammaplasmoparae*, the first bipartite mymonavirus described to date, as well as efforts to identify its host and possible involvement in the studied syndrome.

## 2. Results

### 2.1. Grapevine Symptomatology

**Figure 1:**
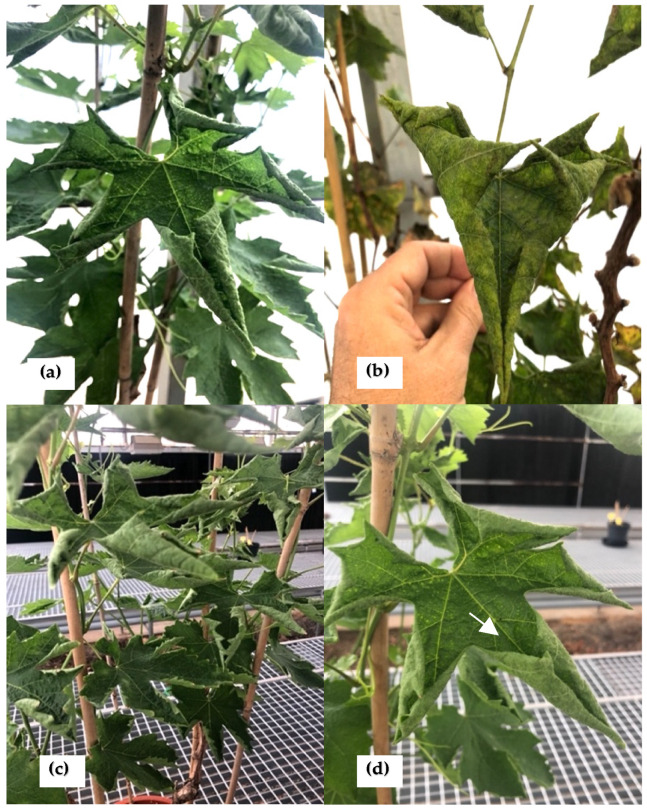
Symptoms observed on the grapevine plants analyzed in the present study: (**a**–**d**) upward leaf rolling and (**d**) leaf puckering. (**a**,**b**) Pin 1. (**c**,**d**) IVIA 49.3.

In 2018, five symptomatic grapevine plants, cultivar Crimson seedless, showing unusual leaf rolling symptoms, were collected from a private vineyard located in Alicante (Spain). The leaves showed a very unusual upward curling phenotype (Figure 1a–d) as well as puckering on the leaf surface (Figure 1d).

The five plants were monitored during three growth seasons (2019–2021) and retained the same unusual symptomatology for the whole period. They eventually suffered a decline and died.

### 2.2. Penicillimonavirus gammaplasmoparae Detection Using HTS

In 2019, total RNA was extracted from leaves from two of the symptomatic grapevines described above (Pin1 and IVIA 49.3) and analyzed using HTS. At the time they were sampled for RNA extraction, the two plants also showed conspicuous grapevine powdery mildew symptoms. HTS analysis yielded 60,597,198 and 49,645,466 reads, respectively, after trimming and quality control steps. A grapevine genome subtraction was then performed by mapping the reads against the grapevine genome and the 8,642,008/5,609,129 remaining reads subjected to de novo assembly. A total of 14,905 (Pin1) and 34,061 (IVIA 49.3) contigs larger than 200 nt were obtained and subsequently analyzed using BLASTN against the RefSeq viral database. The results indicated the presence in the samples of 12 known viruses, including grapevine viruses and mycoviruses (Table 1), none of which has been associated with the observed symptoms.

Among all assembled contigs, the bioinformatic analysis revealed the presence in both samples, Pin1 and IVIA 49.3, of one contig of 6155 nt (deposited in GenBank in July 2022, accession number OP042368, hereafter referred to as RNA2), which showed 99.8% nucleotide identity with the genomic RNA of the recently described *Erysiphe necator* associated negative-stranded RNA virus 2 (NC_077044) and 99.82% nt identity with that of Plasmopara-viticola-lesion-associated mononegaambi virus 3 (NC_076453), indicating these two names to be synonymous. Indeed, this synonymy has been recognized, and following the transition to viral binomial nomenclature, the corresponding virus species has recently been named *Penicillimonavirus gammaplasmoparae* and included in the new genus *Penicillimonavirus* in the family *Mymonaviridae* [22]. Efforts to extend the recovered contig were unsuccessful, consistent with the genome length reported for the other two isolates of, respectively 6178 and 6153 nt.

Interestingly, BLASTX analysis also revealed the presence in both grapevine samples of a smaller contig of 4564 nt (deposited in GenBank in July 2022, accession number OP042367), referred to here as RNA 1, which encodes a protein showing 24.1% amino acid identity with hypothetical protein 2 of Hubei rhabdo-like virus 4 (NC_032783) a member of the family *Mymonaviridae*, raising the possibility that RNA 1 may represent the second genomic segment of a bipartite mymonavirus. Consistent with this hypothesis, BLASTP analysis of the putative codified protein (pII) shows a higher percentage of identity (39.2%) with the nucleoprotein (NP) of the more recently released Magnaporthe oryzae mymonavirus 1 genome (GenBank OL415836).

The analysis of RNA 1 sequence showed the presence of five putative non-overlapping ORFs separated by non-coding intergenic regions: ORF-I encoding a protein of 292 aa with a predicted molecular mass of 31.8 kDa (named pI); ORF-II encoding a protein of 415 aa with a predicted molecular mass of 46.9 kDa (named pII, putative NP); ORF-III encoding a protein of 148 aa with a molecular mass of 17 kDa (named pIII); ORF-IV encoding a protein of 202 aa with a molecular mass of 22.1 kDa (named pIV); and ORF-V encoding a protein of 256 aa with a molecular mass of 29.6 kDa (named pV). ORFs III-V are located in the +2 reading frame, while ORF-I and ORF-II are in the +1 and +3 reading frames, respectively.

Sanger sequencing confirmation of the presence of RNA 1 and RNA 2 in the sampled grapevine tissues and of their sequence was performed using specific primers designed for this study (Supplementary Table S1). The simultaneous presence of both RNAs was also detected using RT-PCR in the three remaining plants showing the unusual symptomatology, using specific primers targeting RNA 1 or RNA 2 (Supplementary Table S1).

*P. gammaplasmoparae* was first identified using HTS [23], and the raw reads used for this discovery are publicly available (Bioproject PRJNA613358) in the Sequence Read Archives (SRAs) of GenBank. In order to validate the co-occurrence of RNA 1 and RNA 2 in the samples in which Plasmopara-viticola-lesion-associated mononegaambi virus 3 genomic RNA was discovered, we performed BLASTN analyses using both RNAs as probes against the relevant SRAs. In 6 out of 16 SRAs, both sequences were found (Supplementary Table S2). On the other hand, in the 10 remaining SRAs, none of the sequences was present. These results indicate a co-occurrence of RNA 1 and RNA 2 and are in agreement with our hypothesis that these two RNA molecules are the two segments of a bipartite genome.

### 2.3. RNA 1 and RNA 2 Share Common Features

**Figure 2:**
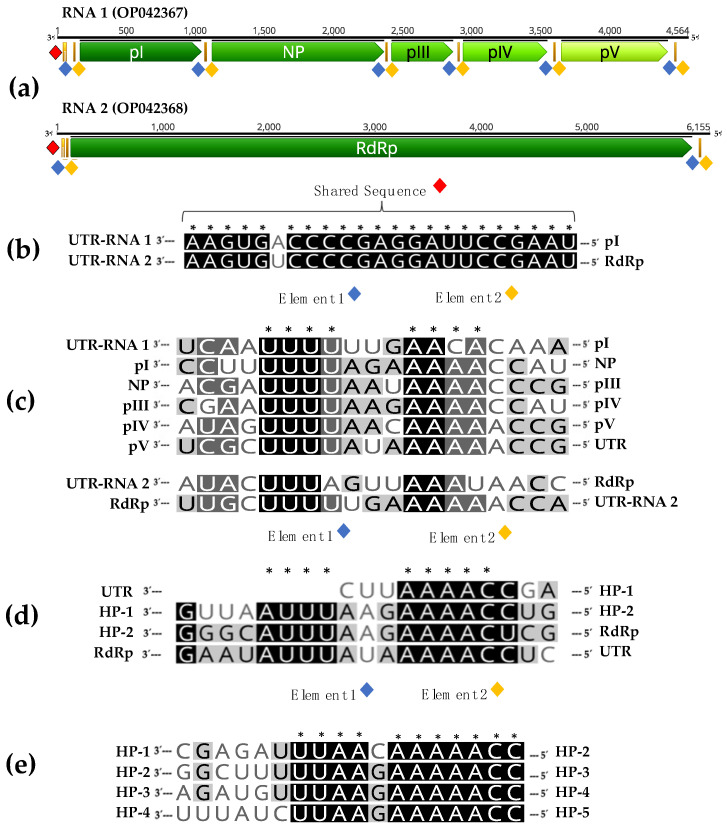
Common genome features in RNA 1 and RNA 2. The genomic structure of RNA 2 and RNA 1 (**a**), as well as the position of conserved elements, are indicated. (**b**) Alignment of the shared sequence (SS) in RNA 1 and RNA 2. (**c**) Alignment of gene junction sequences between ORFs in a 3′-to-5′orientation of RNA 1 and RNA 2 sequences. (**d**) Alignment of gene junction sequences between ORFs in a 3′-to-5′orientation of Hubei rhabdo-like virus 4 (NC_032783). (**e**) Alignment of gene junction sequences between ORFs in a 3′-to-5′orientation of Magnaporthe oryzae mymonavirus 1 (OL415836). (*) Conserved positions.

The association of RNA 1 and RNA 2 is also supported by the finding of common sequence features. Interestingly, both RNAs contain a shared sequence (SS) of 23 nucleotides, with just one single nucleotide polymorphism, in their 3′ untranslated region (3′UTR) (Figure 2b).

In addition, when IGRs and terminal non-coding sequences of both RNAs were analyzed, conserved sequences similar to gene junction sequences of other mymonaviruses, were found in the RNA1 IGRs. Alignment of these conserved sequences in a 3′-to-5′orientation and of similar sequences found in the terminal non-coding regions of both RNAs showed the presence of two conserved elements, named element 1 and element 2, in this study. Element 1 is characterized by the sequence 3′-UUU(U/A)(U/A/G)-5′, while element 2, separated by two nucleotides from element 1, contains the sequence 3′-AA(C/A)(A/U)(C/A)(C/A)-5′.

Elements 1 and 2 are thus present at the intergenic regions between ORFs I-V in RNA 1 and in the 5′ untranslated region (5′UTR) of RNAs 1 and 2 (Figure 2a). Similar elements are present in the IGRs of Hubei rhabdo-like virus 4 (Figure 2d) and Magnaporthe oryzae mymonavirus 1 (Figure 2e), two members of the family *Mymonaviridae*.

### 2.4. Virion Morphology and VANA Analysis

Viral particles were isolated from leaf tissue from the IVIA 49.3 grapevine by differential centrifugation and ultracentrifugation on a sucrose gradient. After gradient fractionation, virion-associated nucleic acids (VANA) were extracted and analyzed for the presence of *P. gammaplasmoparae* by RT-PCR using specific primers targeting RNA 1 or RNA 2 (Supplementary Table S1). Interestingly, both sequences were detected in the same gradient fractions, corresponding to those positive fractions for both RNA 1 and RNA 2, suggesting that the two ssRNAs are either co-encapsidated or separately encapsidated in particles showing similar sedimentation properties (Supplementary Figure S1).

**Figure 3:**
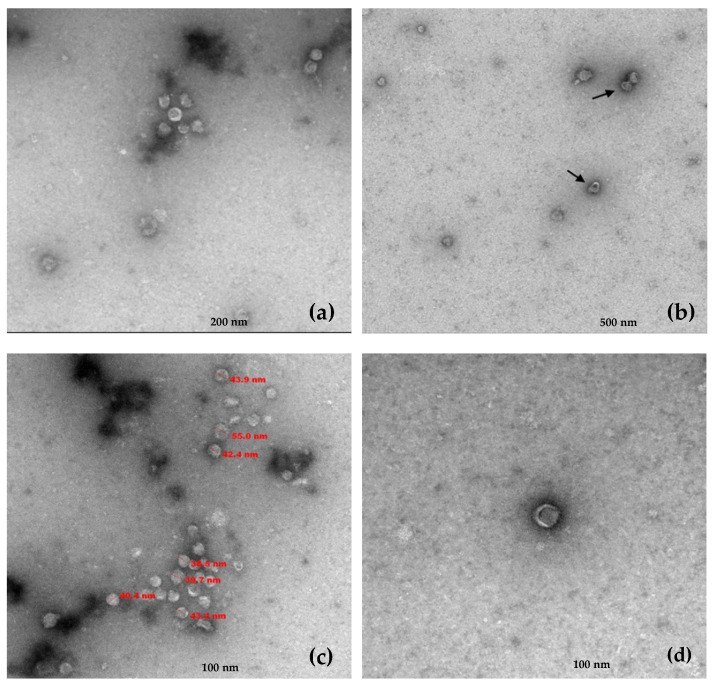
Electron microscopy of viral particles present in positive RNA 1/RNA 2 positive gradient fractions. The scale bar is shown at the bottom of each image. (**a**,**b**) Group of viral particles with similar morphology. (**c**) Size in nm for several particles. (**d**) Virion morphology of a single particle.

Transmission electron microscopy (TEM) performed on the RNA 1/RNA 2 positive gradient fractions revealed the presence of viral particles with a single morphology. These virions exhibited a quasi-spherical morphology with a diameter ranging between 39 and 55 nm and frequently showed crescent or horseshoe-shaped outer zones less dense to electrons (Figure 3).

### 2.5. Phylogenetic Evidence Supporting Penicillimonavirus Bipartite Genome

To further support our findings indicating the bipartite nature of the penicillimonavirus genome, we studied the phylogenetic relationships between several mymonavirus belonging to different genera using the amino acid sequence of the NP, the sole RNA1-encoded protein showing sequence conservation between the different viruses.

During the completion of the present study, several sequences that could correspond to the RNA1 of other Plasmopara-viticola-lesion-associated mononegaambi viruses became available (July 2023; WKE35255, WKE35269, and WKE35277) and were therefore included in the analysis. The sequences of NPs of other members of the following mymonavirus genera were also included: *Hubramonavirus*, *Sclerotimonavirus*, *Auricularimonavirus*, *Lentimonavirus,* and *Plasmopamonavirus*.

**Figure 4:**
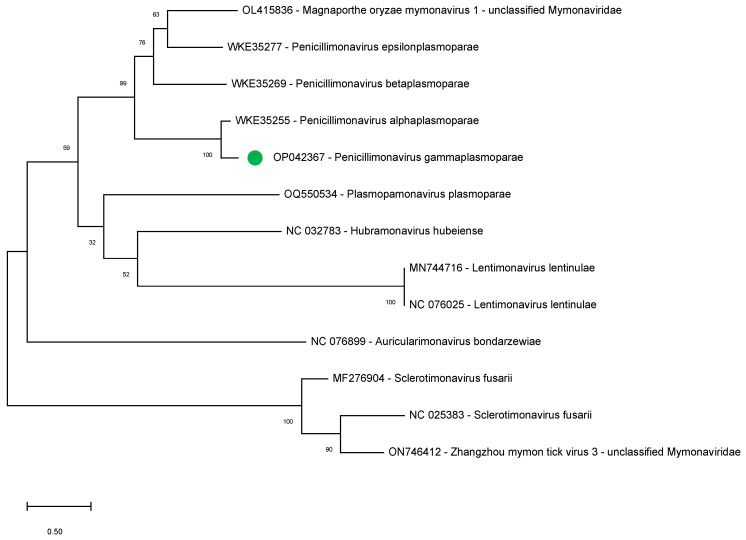
Maximum likelihood phylogenetic tree constructed by MEGA X using the best substitution model, LG + G, with 12 NP aminoacidic sequences of different *Mymonaviridae* members. Accession numbers and assigned genera are indicated. The scale bar shows the number of substitutions per site. Bootstrap percentages (500 resamples) are indicated on the branches.

The results show that the *P. gammaplasmoparae* NP clusters with those of other penicillimonaviruses, strongly supporting the notion of a bipartite genome for all members of this genus (Figure 4).

### 2.6. Efforts to Identify the Host of P. gammaplasmoparae

Plasmopara-viticola-lesion-associated mononegaambi virus 3 has been previously described as field lesions of either downy or powdery grapevine mildew [22,23]. However, the grapevine plants analyzed in the present study did not show signs of *Plasmopara viticola* (downy mildew) infection but were clearly infected by *Erysiphe necator,* the grapevine powdery mildew agent. In order to confirm this, we performed a bioinformatic analysis by mapping the reads obtained for the Pin 1 and IVIA 49.3 plants against the *P. viticola* and *E. necator* genomes. The results of this analysis indicated the presence of *E. necator* in both grapevine plants, with a high coverage of greater than 97% of the fungus genome (Table 2). On the contrary, mapping Pin1 and IVIA 49.3 reads against *P. viticola* reference genome yielded a coverage of less than 5.8% (Table 2). The presence of *E. necator* was also confirmed using RT-PCR using specific primers Uncin144/Uncin511 [24]. Taken together, these results seem to indicate a significant presence of *E. necator* in the two analyzed grapevine plants, suggesting that *P. gammaplasmoparae* may, in fact, be a virus of *E. necator*.

To further study the microbiome of the Pin1 and 49.3 samples, the sequencing reads obtained from these two plants were analyzed using the IDseq-An pipeline [25]. The results obtained confirm the presence of *Erysiphe necator* in both plants (with the signal for *Blumeria graminis*, a *Poaceae* powdery mildew probably also corresponding to *Erysiphe necator*) and the absence of *Plasmopara viticola* in both samples. Raw data from the pipeline results are presented in the Supplementary Tables S3 and S4, respectively. The other fungi identified in the dataset Pin1, with more than 500 reads per million, were *Cladosporium sphaerospermum* and *Wallemia sebi* and the oomycete *Albugo laibachii* (Table S4), and in the case of 49.3, the oomycete *Albugo laibachii.*

It is also important to note that the upward rolling symptomatology observed in the analyzed grapevines is not at all typical of fungi infections such as powdery mildew or downy mildew infection.

## 3. Discussion

This study was initiated investigating the etiology of unusual grapevine symptoms not previously reported, with a conspicuous upward rolling of leaves and eventual plant decline and death. The HTS analysis performed on two of the symptomatic plants showed the presence of several grapevine or fungal viruses not known to be associated with such symptoms, including *P. gammaplasmoparae*, a recently described member of the family *Mymonaviridae*. The results obtained have not allowed us to identify (a) causal agent(s) for the symptoms that initially prompted these efforts. They, however, suggest that *P. gammaplasmoparae* might be a virus of *E. necator*. We cannot, however, rule out the possibility that it infects either grapevine or another unidentified organism associated with the analyzed plants. Unfortunately, the decline and premature death of the grapevine plants have prevented us from performing additional studies in order to explore these possibilities.

In the course of this work, our bioinformatic analyses recovered a 4.6 kb contig with some structural and phylogenetic properties linking it to *Mymonaviridae* members. This contig harbors 5 ORFs, one of which codes for a nucleoprotein (NP) with affinities to those of *Mymonaviridae* members. It also shows a typical genomic organization with conserved IGRs between the various genes that show homologies with those of other family members. On the other hand, no homologies could be identified with any protein in GenBank for the other four hypothetical proteins encoded on this RNA.

Here, we present strong evidence that supports the notion the known *P. gammaplasmoparae* genomic RNA and this novel contig are, in fact, the two RNA segments of a bipartite genome: (i) as outlined just above, the RNA 1 contig has structural and phylogenetic properties linking it to *Mymonaviridae* members (ii) both RNAs (RNA 1 and RNA 2) share conserved terminal regions including a shared sequence of 23 nucleotides nearly totally conserved, a feature typical of the genomic RNAs of viruses with divided genomes [26] (iii) the presence of a single type of viral particles in gradient fractions in which the two molecules co-sedimented and (iv) the systematic presence of the two molecules in the plant samples analyzed by ourselves and by the teams who initially described *P. gammaplasmoparae* as *Erysiphe necator* associated negative-stranded RNA virus 2 and as Plasmopara-viticola-lesion-associated mononegaambi virus 3 [22,23] according to our retrospective data mining. The accumulation of these various lines of evidence makes an extremely strong case for the divided genome hypothesis and, in our mind, essentially rules out the alternate hypothesis that these two molecules are completely unrelated. Therefore, this study represents the complete genome characterization of *Penicillimonavirus gammaplasmoparaee*, a member of the family *Mymonaviridae* with a bipartite genome. We predict this bipartite genome feature as a rule rather than the exception for the genus *Penicillimonavirus*.

During the final redactional stage of this study, a publication also hypothesized the existence of divided genomes for some *Mymonaviridae* on the basis of sequence homologies between *Trichoderma harzianum* mycovirus 1 RNA and a molecule they tentatively associate with *Trichoderma harzianum* mycovirus 2 RNA [27]. They also performed data mining efforts parallel to those reported here, indicating that molecules with homologies with the second RNA they associate with ThMV2 are also systematically identified in metagenomic datasets from which viral segments belonging to *Penicillimonavirus* or *Plasmopamonavirus* genus members were identified, suggesting that a bipartite genome might be a feature of these two genera (although ThMV1, which also appears to be a member of the *Plasmopamonavirus* genus has an undivided genome).

The identification of conserved sequence elements between the two genomic RNAs of *Penicillimonavirus gammaplasmoparaee* and the demonstration that a single type of viral particles is associated with these two molecules provide, we feel, much more convincing elements in favor of a bipartite genome structure than the co-occurrence and homologies brought forward by these authors.

It should be noted that this situation of a group of negative-sense viruses that contains viruses with either a single genomic molecule or a genome divided between two genomic segments is exactly that found in the *Rhabdoviridae* family, with most genera having undivided genomes, but the Dichorhavirus and Varicosavirus genera being characterized by bi-segmented genomes [28].

Overall, this study represents the complete genome characterization of *Penicillimonavirus gammaplasmoparae*, and conclusively demonstrates its bipartite genome nature, a novel feature in the *Mymonaviridae* family. As suggested by Pagnoni et al. [27], we predict this bipartite feature as a rule rather than the exception for the genus *Penicillimonavirus*.

## 4. Materials and Methods

### 4.1. Plant Monitoring and Collection

Five symptomatic grapevine plants, cultivar Crimson seedless, showing upward leaf rolling, were collected from a private vineyard located in Alicante, potted and maintained in a greenhouse, and monitored during three growth seasons.

### 4.2. RNA Purification

Leaf tissue from each symptomatic plant sample was placed in individual plastic bags (Bioreba, Reinach, Switzerland) and ground with extraction buffer (PBS containing 0.2% DIECA and 2% PVP-10) in a 1:5 ratio (w:v). Total RNA was purified from 200 µL of homogenate using the Plant/Fungi total RNA purification kit (Norgen Biotek Corporation, Thorold, ON, Canada) according to the manufacturer’s manual, including DNase treatment using the RNase-Free DNase I Kit (Norgen Biotek Corporation, Thorold, ON, Canada). Purified RNA was quantified with a DeNovix DS-11 spectrophotometer (DeNovix Inc., Wilmington, DE, USA) to determine the concentrations and stored at −80 °C until subsequent analysis.

### 4.3. High Throughput Sequencing

RNA quality control, library construction, and HTS sequencing on a NextSeq 500 platform (paired 2 × 150 nt) were performed at Macrogen Inc. (Seoul, Republic of Korea). Complementary DNA (cDNA) was synthesized from each RNA extraction for library preparation using TruSeq Stranded Total RNA LT Sample Prep Kit (Plant). Library protocol preparation used for it was TruSeq Stranded Total RNA Sample Prep Guide, Part #15031048 Rev.

### 4.4. Bioinformatic Analysis of HTS Data

HTS raw reads data obtained from samples Pin1 and IVIA 49.3 were analyzed as follows: trimming and quality control were performed with CLC Genomics Workbench v.20.0.4 software (Qiagen Bioinformatics, Hilden, Germany). Host genome subtraction was performed using the reference genome GCF_000003745.3 and including mitochondrion (FM179380) and chloroplast (DQ424856) complete organelle genomes. De novo assembly contigs were performed using CLC Genomics Workbench. De novo contigs larger than 200 nt were annotated using BLAST analysis (BLASTN/X) with an e-value cut-off of 10^−3^ against a local virus database. Mapping analysis of the Pin1 and 49.3 reads against the reference the genomes of *P. viticola* (GCA_001695595.3) and *E. necator* (GCA_016906895.1) was performed with the Geneious Prime 2021 software (Biomatters Ltd., Auckland, New Zealand). In addition, metagenomics analysis was performed using the open-source cloud-based platform IDseq v8.2 [25].

### 4.5. RT-PCR and Sanger Sequencing Confirmation

In order to confirm the sequences assembled from HTS data, 22 primer pairs were designed on the RNA 1 and RNA 2 HTS-recovered sequences. Total RNA from the IVIA 49.3 grapevine was used as a template. All RT-PCR reactions were performed on the Applied Biosystems Veriti™ thermal cycler (Applied Biosystems, Foster City, CA, USA) using AgPath-ID One-step RT-PCR kit (Ambion Inc., Austin, TX, USA) on a total volume of 25 µL, containing 3 µL of total RNA as template and 0.5 μM of each primer. RT-PCR protocol consisted of one step of 45 °C for 30 min and 95 °C for 10 min, followed by 45 cycles of amplification (95 °C for 30 s, 49–55 °C for 30 s and 60 °C for 1 min). Amplicons were directly sequenced by Sanger, and sequences were aligned using Mega X software [29].

### 4.6. Viral Particles Isolation by Sucrose Gradient Ultracentrifugation

Viral particle purification was performed from symptomatic plant IVIA 49.3. Twenty grams of leaf tissue were ground with 30 mL of cold (4 °C) extraction buffer supplied with NaCl (150 mM) pH 7.5. First, low-speed centrifugation was carried out at 5000× *g* for 30 min in a Beckman Coulter centrifuge (rotor type JA-20). The supernatant recovered from this low-speed centrifugation was transferred to a new tube and ultracentrifuged at 80,000× *g* for 1 h in a Beckman L-80 ultracentrifuge (rotor type 70.1 Ti). The supernatant was discarded, and the pellet resuspended in 3 mL of gradient buffer (Tris-HCl 150 mM, NaCl 10 mM, and EDTA 1 mM) and loaded on the top of 20 mL of a continuous sucrose gradient (30–70%) prepared in the same buffer and centrifuged 214,000× *g* for 20 h in a Beckman L-80 ultracentrifuge (rotor type TFT 50.38). Gradient fractions of 500 µL were collected using a peristaltic pump system CTP 100 (Thermo Fisher Scientific Inc., Waltham, MA, USA) and a fraction collector FRAC 100 (Pharmacia Biotech, Uppsala, Sweden). RNA from each fraction was extracted and analyzed using RT-PCR as described above, in order to detect the presence of RNA 1 and RNA 2 using primers RNA1-6F and RNA1-580R, as well as primers RNA2-1F and RNA2-554R (Supplementary Table S1).

### 4.7. Transmission Electron Microscopy

Transmission electron microscopy (TEM) was performed from the collected RNA 1 and RNA 2-positive gradient fractions at the microscopy department of Centro de Investigación Principe Felipe (CIPF, Valencia, Spain). Glow Discharge (30 s, 7.2 V, using a Bal-Tec MED 020 Coating System (BalTec AG, Pfäffikon, Switzerland) was applied over Carbon-coated copper grids, and grids were immediately placed on top of sample drops for 10 min. After two brief distilled water washes, grids were contrasted with 1% uranyl acetate for 5 min. Excess fluid was removed, and grids were allowed to dry before examination with a transmission electron microscope FEI Tecnai G2 Spirit (Thermo Fisher Scientific, Waltham, MA, USA). Finally, photomicrographs were obtained under a transmission electron microscope (FEI Tecnai G2 Spirit Biotwin) using a digital camera Morada Soft Imaging System (Olympus, Tokio, Japan).

### 4.8. Sequence Alignment and Phylogenetic Analysis

A phylogenetic analysis was performed on 12 complete NP sequences belonging to different mymonaviruses: OP042367, WKE35255, WKE35269, WKE35277, OQ550534, NC_032783, MN744716, NC_076025, NC_076899, MF276904, NC_025383, ON746412). The multiple alignment was performed using MAFFT (V7.490) implemented in Geneious Prime 2021. The phylogenetic tree was reconstructed in MEGA X [29], using the maximum likelihood algorithm, supported using 500 bootstrap replicates, and selecting the best substitution model computed (LG + G) implemented.

## Figures and Tables

**Table 1:** Viruses were detected in the two symptomatic grapevine plants analyzed using HTS in this study.

Sample Code	De Novo Contigs	Virus Detected ^1^
Pin 1	14,375	GLRaV-1; GLRaV-2; GRSPaV; GFkV; GRVFV; GVA; EnVS-1; EnVS-2; EnVS-3; PsV-F; EnPV-3; *P. gammaplasmoparae*
49.3	34,061	GRSPaV; EnVS-1; EnVS-2; EnVS-3; EnPV-3; *P. gammaplasmoparae*

^1^ Virus detected using BLASTN against the RefSeq database: grapevine leafroll-associated virus 1 (GLRaV-1); grapevine leafroll-associated virus 2 (GLRaV-2); grapevine rupestris stem pitting-associated virus (GRSPaV); grapevine fleck virus (GFkV); grapevine rupestris vein feathering virus (GRVFV); grapevine virus A (GVA); *Erysiphe necator* mitovirus 1 (EnVS-1); *Erysiphe necator* mitovirus 2 (EnVS-2); *Erysiphe necator* mitovirus 3 (EnVS-3); *Penicillium stoloniferum* virus F (PsV-F); *Erysiphe necator* partitivirus 3 (EnPV-3); *Penicillimonavirus gammaplasmoparae* (*P. gammaplasmoparae*).

**Table 2:** Results obtained from the mapping analysis of Pin 1 and IVIA 49.3 reads against the reference genomes of *Plasmopara viticola* and *Erysiphe necator*.

Sample	Fungus Reference Genome (RefSeq)	Number of Mapped Reads	Coverage
Pin1	*Plasmopara viticola* (GCA_001695595.3)	2.567	4.3%
49.3		3.090	5.8%
Pin1	*Erysiphe necator* (GCA_016906895.1)	356.145	97.3%
49.3		398.428	97.8%

## Data Availability

All data supporting the results and conclusions of the study are contained within the article. Generated sequences have been submitted to GenBank. HTS reads covering the full-length genomes recovered in this study are available upon request.

## Supplementary Materials

The following are available online at https://www.mdpi.com/article/10.3390/plants12183300/s1, Table S1: Primers used for detection and Sanger sequencing of *Penicillimonavirus gammaplasmoparae* RNA 1 and RNA2 genomic segments, Table S2: Results of the BLASTN analysis performed for detection of *P. gammaplasmoparae* RNA1 and RNA2 detection in SRAs (Bioproject PRJNA613358), Table S3: Metagenomic CZ IDSeq analysis results for sample Pin1, Table S4: Metagenomic CZ IDSeq analysis results for IVIA 49.3, Figure S1: Co-sedimentation of *P. gammaplasmoparae* RNA1 and RNA2 in a sucrose gradient.
