# Supplementary material for: Complete Genome Characterization of Penicillimonavirus gammaplasmoparae, a Bipartite Member of the Family Mymonaviridae
Source: Plants (Basel). 2023 Sep 18;12(18):3300. doi: 10.3390/plants12183300 (PMC10538141; doi:10.3390/plants12183300)
Supplement: Supplementary file 1 [file plants-12-03300-s001.zip › Supplementary Figure S1.pdf]

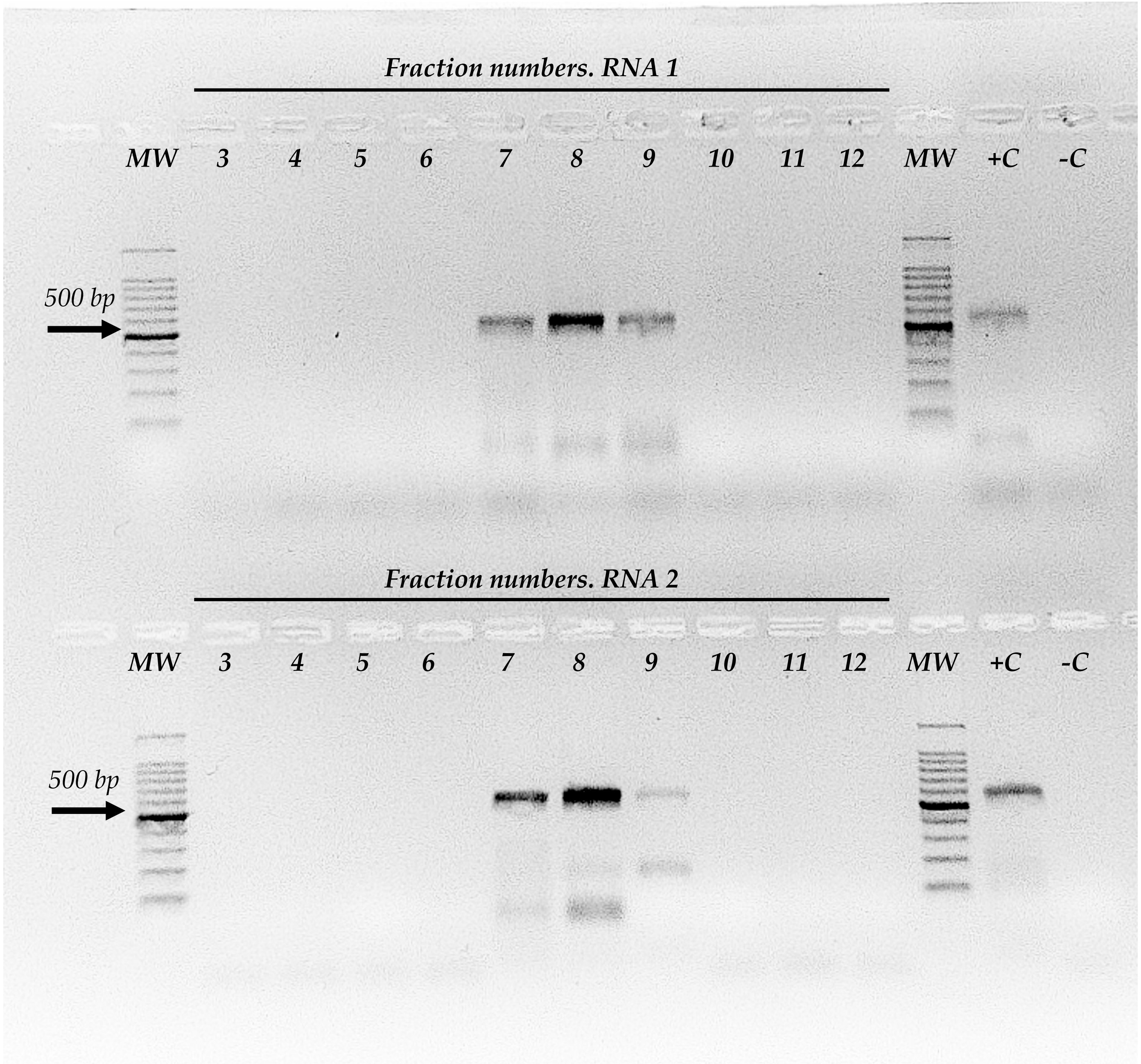

Figure S1: Co-sedimentation of *P. gammaplasmoparae* RNA1 and RNA2 in a sucrose gradient.
