# Supplementary material for: Complete Genome Characterization of Penicillimonavirus gammaplasmoparae, a Bipartite Member of the Family Mymonaviridae
Source: Plants (Basel). 2023 Sep 18;12(18):3300. doi: 10.3390/plants12183300 (PMC10538141; doi:10.3390/plants12183300)
Supplement: Supplementary file 1 [file plants-12-03300-s001.zip › Supplementary Table S1.pdf]

Table 1. Primers used for detection and Sanger sequencing of Penicillimonavirus gammaplasmopara RNA 1 and RNA2 genomic segments. The target region and the amplified size (nt) are indicated.

Penicillimonavirus gammaplasmopara	Primer name	Primer sequence (5′-3′)	Target	Product size (nt)
RNA_1 (OP042367)	C-II-1F_Detection	GTC CGC GCA AAA ATA AGA TAC	3′-UTR/pV	554
	C-II-554R_Detection	AGC ACG TTC AGA TTC TGA GTG		
	C-II-17F	GAT ACA GAC AGA CTA ACT ATG ATG AAG	3′-UTR/pV	538
	C-II-554R_Detection	AGC ACG TTC AGA TTC TGA GTG		
	C-II-479F	ACT CTC ATA GTC ACT TTT GTC G	E2/pV	478
	C-II-957R	GAA CCT CAT CGA TAA GTA CAA CC		
	C-II-1349F	GGA TCA GTA GGG TGA AGA GAA G	pIV/pV	568
	C-II-1917R	TCA GTA TCT ATG AGT TAC GGA AAG A		
	C-II-1796F	AGT TCC AGA TAG TTT CTC TCT CC	pII/pIII	599
	C-II-2395R	GAA ACT CTC TTT TGA CCC TTG C		
	C-II-2769F	AGG AAT CTT GTA GGT GTC AAT G	E2/pII	709
	C-II-3478R	CTT GTA CCA CCG CTC CAG		
	C-II-3321F	GTT GAT GGT GCG AGA ATA T	pI/pII	417
	C-II-3738R	ATA CTC AAC TGA TGG ATC ACG TT		
	C-II-3406F	TGC TCG ACG TCC TTG ATC AG	pI/pII	662
	C-II-4068R	AAC AAA TGA AGC GTG GCC T		
	C-II-3716F	AAC GTG ATC CAT CAG TTG AGT AT	pI	380
	C-II-4096R	AGT CTA GTC AAG CAA GAT GAG GA		
	C-II-3999F	ACG TTC GTG TTT ACA AAA GGC	5′-UTR/pI	531
	C-II-4530R	AAA GTG CGA AAG TGA CCC		
	C-II-3999F	ACG TTC GTG TTT ACA AAA GGC	5′-UTR/pI	565
	C-II-4564R	CGC AAA AAT AAG ATA TAG ACA GAC GA		

	C-I-6F_Detection	GAG TGA CAG TGG TTT TTT CAA AA	3′UTR/RdRp	574
	C-I-580R_ Detection	CGC ATC ACT CAG TAG AAC TTA TC		
	C-I-981F	GTC AGC CAG TCC TCC TAA	RdRp	575
	C-I-1556R	TTT GCT GGT GAG GAC ATA AC		
	C-I-1495F	ATG GCC CTA CAA CCG AGA G	RdRp	542
	C-I-2037R	CGC ATC TAA AGA GTG TGT TTC A		
	C-I-1938F	GTA TCC TCG TGT ATC AGC TAA C	RdRp	687
	C-I-2625R	GGA ACA CGG CTA CAA GTT AG		
	C-I-2498F	GAG TGT AGG CTA TGT TAT CTA AGA G	RdRp	632
	C-I-3130R	ATA TCT CTC CGC TAC TCA ACC		
RNA_2 (OP042368)	C-I-3433F	GGC AGT AAC AAT GCC ATC AAG A	RdRp	581
	C-I-4014R	GGG AGG GTT TGA AGG ACT GAA		
	C-I-3911F	ACG AGG TGA TTA TAT TGT TAA GCA T	RdRp	632
	C-I-4543R	AGC TTC TCA ACA GAC GGA ACT T		
	C-I-4427F	GCT TGA ATT CTC GTT CCT TTG	RdRp	683
	C-I-5110R	CTG CAT ATA CGA GAA TGA TTG TCA		
	C-II-5012F	TGG CAC ACA ATT TTG TCA CAC TT	RdRp	602
	C-II-5613R	GTT AGT TGA AGA AGC CCT GTG T		
	C-I-5527F	TTA TTT AAA CGA TTA GAC ACA CCT TTG	5′-UTR/RdRp	541
	C-I-6068R	ATT ATT CGACGCG ATT GTG CGG T		
	C-I-5527F	TTA TTT AAA CGA TTA GAC ACA CCT TTG	5′-UTR/RdRp	567
	C-I-6094R	GCA CAC CAA AGG AAG GAA ATA CAG		
