# Supplementary material for: Complete Genome Characterization of Penicillimonavirus gammaplasmoparae, a Bipartite Member of the Family Mymonaviridae
Source: Plants (Basel). 2023 Sep 18;12(18):3300. doi: 10.3390/plants12183300 (PMC10538141; doi:10.3390/plants12183300)
Supplement: Supplementary file 1 [file plants-12-03300-s001.zip › Supplementary Table S2.pdf]

Table S2. Results of the BLASTN analysis performed for detection of *P. gammaplasmopara* RNA1 and RNA2 detection in SRAs (Bioproject PRJNA613358). Number of reads, coverage of RNA1/RNA2 molecules and E-value is shown.

Bioproject PRJNA613358		BLASTN of RNA_1 (OP042367) against each SRA				BLASTN of RNA_2 (OP042368) against each SRA			
SRA	Sample name	Nº reads	Coverage	Identity	E-value	Nº reads	Coverage	Identity	E-value
SRX7966246	DMGA	>5000	56,3%	99.5%	$3x10^{-16}$	>5000	48,2%	98.9%	$3x10^{-16}$
SRX7966247	DMGB	902	98.1%	96.7%	$3x10^{-42}$	2095	99,8%	79%	$3x10^{-42}$
SRX7966254	DMGC	>5000	97%	100%	$3x10^{-42}$	>5000	99,8%	87.1%	$3x10^{-42}$
SRX7966255	DMGD	>5000	98.1%	98.4%	$2x10^{-42}$	3292	99.8%	89.5%	$2x10^{-42}$
SRX7966256	DMGE	>5000	95.0%	100%	$2x10^{-42}$	>5000	99.1%	100%	$3x10^{-42}$
SRX7966257	DMGF	-	-	-	-	-	-	-	-
SRX7966258	DMGG	>5000	96.0%	99.8%	$3x10^{-42}$	>5000	99.4%	99.9%	$3x10^{-42}$
SRX7966259	DMS1	-	-	-	-	-	-	-	-
SRX7966253	DMS10	-	-	-	-	-	-	-	-
SRX7966260	DMS2	-	-	-	-	-	-	-	-
SRX7966261	DMS3	-	-	-	-	-	-	-	-
SRX7966248	DMS4	-	-	-	-	-	-	-	-
SRX7966249	DMS5	-	-	-	-	-	-	-	-
SRX7966250	DMS6	-	-	-	-	-	-	-	-
SRX7966251	DMS8	-	-	-	-	-	-	-	-
SRX7966252	DMS9	-	-	-	-	-	-	-	-
